# Evaluation of a collaborative ambulatory orthopedic care program for patients with hip and knee osteoarthritis: a comparative observational cohort study

**DOI:** 10.1186/s12891-022-05697-9

**Published:** 2022-08-03

**Authors:** Angelina Müller, Olga A. Amberger, Anastasiya Glushan, Claudia Witte, Renate Klaaßen-Mielke, Burkhard Lembeck, Martin Beyer, Ferdinand M. Gerlach, Kateryna Karimova

**Affiliations:** 1 Institute of General Practice, Goethe-University, Theodor-Stern-Kai 7, 60590 Frankfurt, Germany; 2 aQua, Institute for Applied Quality Improvement and Research in Health Care, 37073 Goettingen, Germany; 3 Institute of Medical Informatics, Biometry and Epidemiology, Ruhr University, 44789 Bochum, Germany; 4 Joint Practice for Orthopaedic and Trauma, Hindenburgstr. 7/1, 73730 Ostfildern, Germany

**Keywords:** Osteoarthritis, Health service utilization, Collaborative care, Multivariable analysis, Hospitalization risk

## Abstract

**Background:**

In 2014, the novel orthopedic care program was established by the AOK health insurance fund in southern Germany to improve ambulatory care for patients with musculoskeletal disorders. The program offers extended consultation times, structured collaboration between general practitioners and specialists, as well as a renewed focus on guideline-recommended therapies and patient empowerment. The aim of this study was to assess the impact of the program on health service utilization in patients with hip and knee osteoarthritis (OA).

**Methods:**

This retrospective cohort study, which is based on claims data, evaluated health service utilization in patients with hip and knee OA from 2014 to 2017. The intervention group comprised OA patients enrolled in collaborative ambulatory orthopedic care, and the control group received usual care. The outcomes were participation in exercise interventions, prescription of physical therapy, OA-related hospitalization, and endoprosthetic surgery rates. Generalized linear regression models were used to analyze the effect of the intervention.

**Results:**

Claims data for 24,170 patients were analyzed. Data for the 23,042 patients in the intervention group were compared with data for the 1,128 patients in the control group. Participation in exercise interventions (Odds Ratio (OR): 1.781; 95% Confidence Interval (CI): 1.230–2.577; *p* = 0.0022), and overall prescriptions of physical therapy (Rate Ratio (RR): 1.126; 95% CI: 1.025–1.236; *p* = 0.0128) were significantly higher in the intervention group. The intervention group had a significantly lower risk of OA -related hospitalization (OR: 0.375; 95% CI: 0.290–0.485; *p* < 0.0001). Endoprosthetic surgery of the knee was performed in 53.8% of hospitalized patients in the intervention group vs. 57.5% in the control group; 27.7% of hospitalized patients underwent endoprosthetic surgery of the hip in the intervention group versus 37.0% in the control group.

**Conclusions:**

In patients with hip and knee OA, collaborative ambulatory orthopedic care is associated with a lower risk of OA-related hospitalization, higher participation in exercise interventions, and more frequently prescribed physical therapy.

## Background

Osteoarthritis (OA) of hip and knee affects a high percentage of the worldwide population and is ranked 11th in terms of years lived with disability [1]. Its worldwide prevalence is continually monitored in the Global Burden of Disease study [1, 2] and effective management strategies for OA are recommended [2].

In Germany, information on prevalence rates is lacking [3] and available data are generally based on self-report [4, 5]. The self-reported prevalence of OA is 15—31%, whereby higher rates are associated with older age [6]. Another peculiarity of the German health care system is that a distinction is made between primary and secondary care for patients with OA of hip and knee. Although treatment of patients with OA often involves orthopedists, they are not generally responsible for providing primary care services, which, as a result of the gatekeeping system, are mainly provided by general practitioners (GPs). Since OA is a chronic condition, lifestyle and working conditions play a crucial role in determining the course of the disease [7] and effective disease management strategies are required.

In our study we describe a novel health care program for OA patients in Germany that aims to promote cooperation between primary and specialist care and strengthen patient-centered consultations, with the aim of improving orthopedic care for OA outpatients.

The orthopedic care program is integrated into a well-established GP-centered care program [8, 9]. The GP-centered care program in the federal state of Baden-Wuerttemberg was launched in 2004 and is based on a structured disease management model that promotes collaboration between health care providers that would otherwise work independently of one another [10]. In GP-centered care, the GP is the gatekeeper and coordinates care. Other aspects of the program are prompt access to specialized care, continuity of care, and data-driven quality improvement [11]. In order to prevent fragmentation of care, specialist programs were developed that concentrate on using a stepped approach that incorporates the chronic care model [11]. In Germany, the introduction of such programs has been facilitated by the creation of a legal framework aimed at improving patient management in ambulatory care [12].

The orthopedic care program follows the stepped-care approach and reflects a renewed focus on GP-centered care [12, 13]. Core features include such elements of managed care as the regulation of healthcare provision, selective contracting with healthcare providers, and improved access to healthcare for orthopedic patients. Other elements of the program (see Table 1) include the promotion of guideline-recommended care, and especially of non-drug therapies. It also aims to reduce unnecessary diagnostic procedures (diagnostic imaging), and ensure adherence to quality requirements, continuous quality improvement, participation in peer group training sessions, and the use of care pathways to coordinate care. Communication between GPs and orthopedists that participate in the program is standardized, and the exchange of important clinical information is obligatory. Other specialist programs are offered for cardiology, gastroenterology, neurology and psychiatry, urology, diabetology, nephrology and pneumology. Studies of similarly structured specialist programs, such as the cardiology care program, have shown the programs to be associated with reduced hospitalization risk and mortality in their respective groups of patients [12].

**Table 1:** Special characteristics of the orthopedic care program and additional care provided to intervention group participants

**Components of the orthopedic care program (a)**
Promotion of guideline-recommended care
Defragmentation of health-care
Focus on chronic diseases due to rising life expectancy
Incentives to repeat consultations in critical clinical situations and to ensure pharmacotherapy is evidence-based
Morbidity-adapted reimbursement
Patient safety enhanced via multidisciplinary, structured and biopsychosocial-preventive care
Continuous data-driven quality improvement
Participation in clinical peer group training sessions, e.g. in drug therapy
Coordinated care pathways standardize communication between general practitioners and orthopedists
Provision of patient education and emphasis on nationwide disease management programs
**Components of orthopedic care program provided in addition to usual care (b)**
Participating specialists take part in regular and obligatory advanced training
Coordinated care pathways standardize cooperation between general practitioners and specialists (orthopedists)
Appointment within two weeks (in urgent cases appointment on the same day)
Doctor’s office waiting times may not exceed 30 min. Consultations also provided in the evenings
Special health-care services such as AOK-Sports and AOK-ProReha (additional services for patients requiring rehabilitation after surgery)

(a) https://www.aok.de/pk/bw/inhalt/facharztprogramm-orthopaedie/

(b) https://www.aok.de/pk/fileadmin/user_upload/AOK-Baden-Wuerttemberg/05-Content-PDF/aokbw-facharztprogrammf-flyer-englisch.pdf

Based on an evidence-based and above all biopsychosocial assessment, the program focuses on the reduction of over-, under- and incorrect treatment for musculoskeletal diseases and includes structured motivational and preventive advice to promote health by strengthening self-management [14].

In order to promote such frontline interventions for OA as lifestyle changes, exercise, and self-management [15], the program focuses in particular on an initial assessment of patients’ biopsychosocial backgrounds and an individualized therapy [7]. Since patient empowerment [16], self-help and patient-driven treatments [17] are considered cornerstones of successful chronic care for patients with OA, the purpose of the orthopedic care program is to enhance the ue of these interventions by fusing the orthopedic care program with GP-centered care.

By focusing on patients with OA of the hip and knee, the aim of this study was to evaluate the collaborative orthopedic care program in terms of OA-related hospitalization, participation in exercise interventions, prescription rates for physical therapy, coordination and continuity of care and endoprosthetic surgery rates.

## Methods

We carried out a retrospective observational comparative cohort study based on administrative data provided by the statutory health insurance fund ‘Allgemeine Ortskrankenkasse’ (AOK), in the state of Baden-Wuerttemberg, Germany, for the years 2014 to 2017. In the observation period, Baden-Wurttemberg had about 11.1 million inhabitants, of whom 5.1 million were insured by the AOK [18]. The AOK is the largest health fund in the state, providing insurance to about 40% of the insured population [19].

Based on GP-centered care, the AOK selectively negotiated contracts with specialist care providers. One of these contracts covered a program for orthopedic health-care. By 2016, about 550 orthopedists and orthopedic surgeons providing outpatient care, and 350,000 insured persons, had enrolled in the collaborative orthopedic care program [20].

In our study we performed a secondary analysis of the effectiveness of this program.

### Intervention

#### Collaborative ambulatory orthopedic care program

In 2014, the orthopedic collaborative care program evaluated in this study was launched for outpatients with non-specific and specific back pain, OA of hip and knee, osteoporosis and rheumatoid arthritis in Baden-Wurttemberg, Germany [14]. The program is a structured program based on a selective contract for orthopedists and orthopedic surgeons. Further details and the legal framework can be found in the German Social Code, Book 5 (SGB V) §73c. The contracting partners in the program are AOK, Bosch BKK (health insurance fund), MEDIVERBUND AG, BVOU (Professional Association of Orthopedic Specialists and Orthopedic Surgeons), BNC (Federal Association of Surgeons), BDRh (Federal Association of German Rheumatologists) and Rheumexperten BW eG (an association of practices specializing in rheumatology). Participation is voluntary for both doctors (orthopedists and orthopedic surgeons in private practice) and patients. However, participants must have previously enrolled in the GP-centered care program in which the orthopedic program is embedded [8, 11]. The GP-centered care program is available throughout the study region. The medical specialist programs are not only offered to patients with musculoskeletal disorders, but are also available for other diseases [12]. Patients wishing to enroll in them are required to first consult their GP, who will refer them to a specialist where necessary, and coordinate further treatment. To ensure no information is lost, participating doctors share clinical and other patient information with their colleagues electronically. Compared to usual care, patients profit from shorter waiting times for appointments and higher continuity of care [8].

The main goals of the orthopedic care program are to implement guideline-oriented care for OA patients in an outpatient setting by, for example, enhancing biopsychosocial anamneses and strengthening motivational consultations. The use of financial incentives (provided by the health insurance fund) for extended consultation times is intended to encourage physicians to provide detailed information and promote patient self-management. Orthopedists are encouraged to perform evidence-based medicine and to empower their patients. The increased use of non-pharmacological and non-surgical treatments are considered cornerstones of the program. Thorough awareness of individual biopsychosocial risk factors is expected to facilitate implementation and adherence to individual therapeutic plans.

Regular clinical peer group meetings and continuous data-driven quality improvements are features of the program’s extended quality management.

### Participants

Participants were selected according to insurance status, relevant diagnoses, and ICD-10-M codes. Patients that fulfilled the following criteria were included: diagnosis of hip and knee OA (ICD-code M16.0–16.7 or M17.0–17.5), uninterrupted health insurance in the period under review, resident in Baden-Wuerttemberg and aged over 18 years. We included OA patients that were diagnosed with OA in the first or second quartile of 2016 and had additionally been diagnosed with OA in 2014 or 2015 (irrespective of whether they had been diagnosed with OA prior to 2014). The diagnosis of any physician involved in the care of the patient was considered valid. Baseline characteristics of patients and relevant comorbidities were assessed during a pre-observation period in 2015.

The intervention group included patients that were enrolled in the orthopedic care program throughout the observation period, and that had consulted an orthopedist or orthopedic surgeon enrolled in the program at least once during that time. Contact with the orthopedist was operationalized using the corresponding billing code. The control group included patients that were not enrolled in either the orthopedic program or the GP-centered care program, and that had consulted a non-participating orthopedist or orthopedic surgeon at least once. The control group received usual care, meaning care provided by a non-participating orthopedist or orthopedic surgeon. In Germany, patients can seek care from an orthopedist or orthopedic surgeon in private practice either directly, or upon referral by a GP.

Patients that opted out of the orthopedic program or the GP-centered care program during the observation period were excluded from our study. Moreover, patients that had only enrolled in GP-centered care and not in the orthopedic care program, were also excluded.

We also excluded patients that had enrolled in the orthopedic program but had consulted a non-participating orthopedist or orthopedic surgeon. Furthermore, to reduce contamination to a minimum, we excluded both patients that enrolled in GP-centered care or the orthopedic care program during the observation period, and patients that sought care from a participating orthopedist but had not enrolled in the orthopedic care program.

**Fig. 1:**
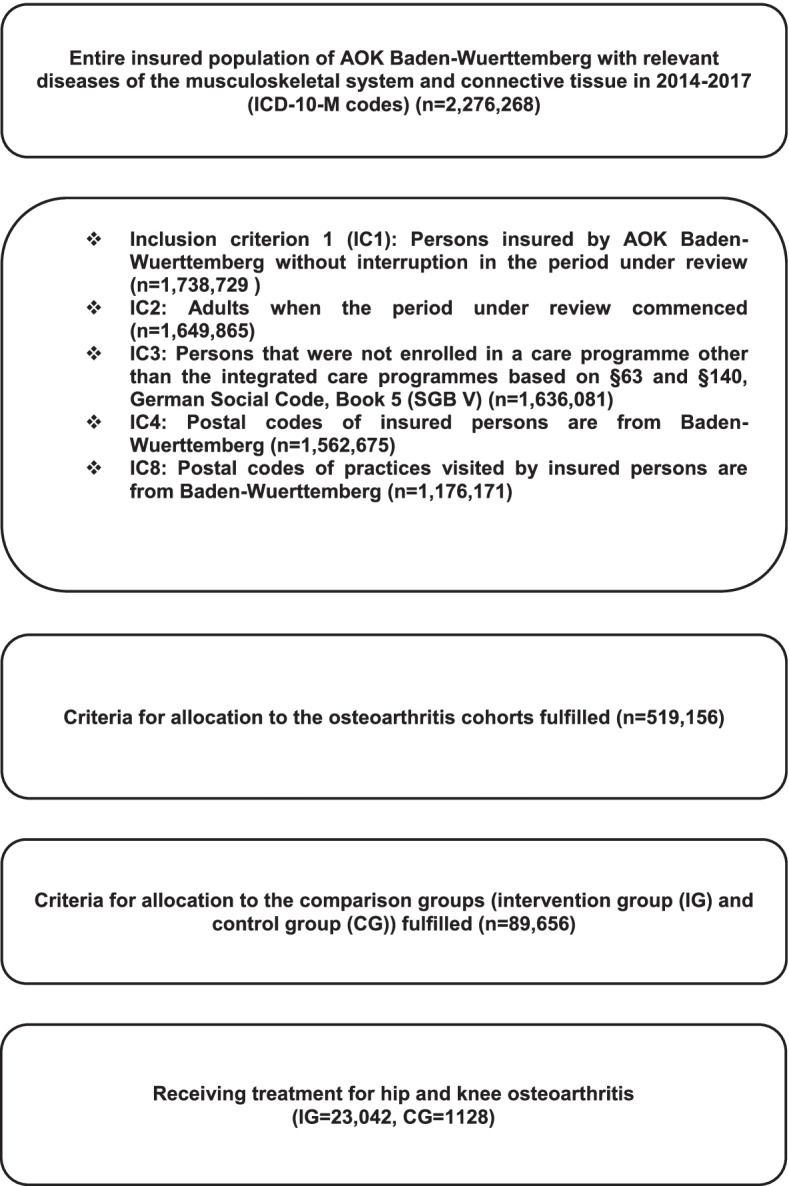
Inclusion criteria for study groups

A detailed description of the study population and inclusion and exclusion criteria can be found in Fig. 1. Reports on this observational study were prepared in accordance with the STROBE Statement and the German reporting standard for secondary data analysis (STROSA) [21].

### Outcomes

Outcomes were assessed in the observation period, which lasted from July 1, 2016, to December 31, 2017 (six quartiles). The primary outcome was OA-related hospitalization. Hospitalization due to OA was defined as in-hospital medical service use by a patient that had been diagnosed with OA. Other outcomes included participation in exercise interventions, prescribed physical therapy, coordination and continuity of care, as well as endoprosthetic surgery rates among hospitalized patients. Data was only available for exercise interventions organized by the AOK health fund and reflect actual participation in exercise interventions by patients. Exercise interventions included local courses in, for example, resistance training, lower back training, walking, balance and agility training, and exercises for hip and knee. They were offered in the form of institution-based group exercise.

Physical therapy included manual therapy, heat therapy, electrotherapy, massages, traction treatments and medical baths. Outcomes refer to the number of prescriptions for physical therapy and do not necessarily correspond to the amount of physical therapy actually received by the patient. Physical therapy was identified using specific procedure codes, which include all procedures that can be prescribed for OA.

Coordination and continuity of care, referred to here as collaborative care, were assessed by measuring the number of quartiles in which contact to an orthopedist occurred without a prior GP referral (uncoordinated care), and the overall number of quartiles in which an orthopedist was consulted.

Endoprosthetic surgery rates were calculated using the corresponding OPS-code for hip/knee replacement. All measures were based on routinely available claims data. The study is part of an evaluation of the entire orthopedic care program, in which further outcomes are being assessed.

### Covariables

The selection of covariables was based on medical considerations, current literature [22–25] and availability in the claims data. The Charlson Comorbidity Index (CCI) score [26] was used in combination with the nursing level (level of need of nursing care) to adjust for comorbidities and frailty. High prevalence entities, and common comorbidities [6] that were not already covered in the CCI, were also included. The following covariables were chosen for the model: age, sex, participation in disease-management-programs, CCI, nursing level, cardiovascular diseases, diabetes, stroke, malignoma, obesity, depression, psycho-social risk factors, burn-out, smoking and history of OA-related health-care utilization. The selection of covariables was defined at baseline.

### Statistics

Initially, all outcome and influencing variables were described descriptively. The number of non-missing values, mean, standard deviation, median, 1st and 3rd quartile, minimum and maximum were specified for continuous variables, and absolute and relative frequencies calculated for categorical variables.

A generalized linear regression model was used to analyze the intervention effect for each outcome variable. As independent factors group variable for the intervention and other covariates (potential confounders) were included in the fixed effects model. Depending on the outcome variable, logistic and negative-binomial regression models were used. Results were presented as odds ratios (OR) for binary variables and rate ratios (RR) for count variables, and with 95% confidence intervals (CI). We considered two-sided *p*-values and labelled *p*-values < 0.05 as significant.

All descriptive and comparative analyses were carried out in accordance with Good Practice in Secondary Data Analysis [27]. using SAS (version 9.4) and IBM SPSS Statistics (version 25).

## Results

We included 23,042 patients in the intervention group and 1,128 patients in the control group. Mean age in the intervention group was 68.9 ± 11.2 years and 71.7 ± 11.4 years in the control group. Overall, the prevalence of diabetes, malignoma, smoking and depression was higher in patients assigned to the intervention group, whereas those in the control group showed higher rates of psychic risk factors and burn-out. The CCI was higher in the intervention group (2.2 ± 2.2 vs. 1.6 ± 1.9). Further patient characteristics are displayed in Table 2.

**Table 2:** Baseline characteristics/confounders for study population

**Baseline characteristics**	**Usual care** *N* = 1,128	**Collaborative care** *N* = 23,042	***p*-value**^a^
**age (mean [SD])**	71.7 [11.4]	68.9 [11.2]	< 0.001
**sex (female)**	66.0%	66.3%	0.841
**level of care**	6.9%	3.6%	< 0.001
**CCI (mean [SD])**	1.6 [1.9]	2.2 [2.2]	< 0.001
**participation in disease-management programme for type 2 diabetes**	16.0%	24.4%	< 0.001
**cardiovascular comorbidities**	77.2%	76.7%	0.669
**type 2 diabetes**	27.2%	29.5%	0.099
**stroke and other cerebrovascular diseases**	2.2%	2.8%	0.214
**malignoma**	14.6%	16.0%	0.234
**obesity**	27.9%	28.9%	0.467
**depression**	21.9%	34.3%	< 0.001
**smoking**	3.9%	4.8%	0.164
**psychosocial risk factors**	2.0%	1.4%	0.109
**burn-out**	5.7%	5.5%	0.850
**somatoform disorders**	14.3%	21.3%	< 0.001
**MDPTO score (mean [SD])**^b^	3.7 [1.8]	4.1 [1.8]	< 0.001

^a^t-test for count and continuous variables, chi-square-test for binary variables

^b^MDPTO score: The musculoskeletal-disorder-prior to observation (MDPTO)-score is a non-standardized score we developed and used in the model to approximate level of musculoskeletal disease prior to the observation period. The score takes into account the following factors during the pre-observation period: hospitalisation related to musculoskeletal disorders, diagnosis of musculoskeletal disease, sickness certificate for musculoskeletal disorders, prescription of physical therapy or aids and appliances and prescription of opioids

### OA-related hospitalization

Hospitalization rates were lower in the intervention group than in the control group (2.5 and 6.5% respectively). The results of the multivariable analysis showed that patients in the intervention group had a lower chance of OA-related hospitalization (OR 0.375; 95% CI 0.290–0.485; *p* < 0.0001).

### Exercise interventions and physical therapy

Rates of participation in an exercise intervention were 5.1% in the intervention group and 2.7% among controls. Multivariable analysis showed that patients in the intervention group were more likely to participate in exercise interventions than patients in the control group (OR 1.781; 95% CI 1.230–2,577; *p* = 0.0022). Physical therapy prescriptions (including manual therapy, heat therapy and massages) were higher in the intervention group (2.4 ± 3.6 vs. 2.0 ± 3.5; RR 1.126; 95% CI 1.025–1.236; *p* = 0.0128), with 57.7% of patients in the intervention group receiving at least one physical therapy prescription and 45.9% in the control group.

### Collaborative care

In the intervention group, multivariable analyses of coordination and continuity of care demonstrated that the risk of uncoordinated contacts (e.g. appointments with orthopedists without a prior GP-referral) were significantly reduced (0.3 ± 1.1 vs. 3.5 ± 5.4; RR 0.085; 95% CI 0.068–0.107; *p* < 0.0001), but the overall number of quartiles during which patients had consulted an orthopedist was higher (2.5 ± 1.9 vs. 1.6 ± 1.7; RR 1.527; 95% CI 1.445–1.613; *p* < 0.0001).

### Endoprosthetic surgery

Results of the descriptive analysis of endoprosthetic surgery performed during OA-related hospital stays are displayed in Table 3. OPS-Codes 5–820 and 5–821 were used for hip replacement or repeat hip replacement, and OPS-Codes 5–822 and 5–823 for knee replacement or repeat knee replacement. We decided not to perform multivariable analysis for endoprosthetic surgery due to the low sample sizes.

**Table 3:** Descriptive statistics and results of the multivariable analysis

**Outcome**	**Usual care Rate [%] *N* = 1,128**	**Collaborative care Rate [%] *N* = 23,042**	**RR/OR**^a^	**95%-confidence intervall**		***p*-value**
Overall rate of OA related hospitalization	6.5%	2.5%	0.375	0.290	0.485	< 0.001
Number of physical therapy prescription (mean [SD])	2.0 [3.5]	2.4 [3.6]	1.126	1.025	1.236	0.013
Participation in exercise intervention	2.7%	5.1%	1.781	1.230	2.577	0.002
Number of quartiles with orthopaedist visits (mean [SD])	1.6 [1.7]	2.5 [1.9]	1.527	1.445	1.613	< 0.001
Number of uncoordinated orthopaedist contacts (mean [SD])	*N* = 746	*N* = 19,281	0.085	0.068	0.107	< 0.001
	3.5 [5.4]	0.3 [1.1]				
Overall rate of hip replacement or repeat hip replacement	*N* = 27	*N* = 159				
	2.4%	0.7%				
Overall rate of knee replacement or repeat knee replacement	*N* = 42	*N* = 309				
	3.7%	1.3%				
Hip replacement or repeat hip replacement among hospitalised patients	*N* = 73	*N* = 574				
	57.5%	53.8%				
Knee replacement or repeat knee replacement among hospitalised patients	*N* = 73	*N* = 574				
	37.0%	27.7%				

^a^For count variables we estimated the rate ratio (RR), for binary variables the odds ratio (OR)

## Discussion

We studied the collaborative orthopedic care program in the federal state of Baden-Wuerttemberg, Germany, and observed that a reduced risk of hospitalization was associated with enrolment in the program. Since continuity and coordination of care were significantly higher in patients receiving collaborative orthopedic care, the implementation of the program can be considered successful. This should be taken into account when examining other aspects and effects.

Previous epidemiological studies in Germany have assessed health-care utilization prior to total joint replacement in OA patients [28] and analyzed administrative data of elderly OA patients [6]. To our knowledge, our study is the first epidemiological study in Germany to focus on health-care utilization of OA patients enrolled in a structured health-care program.

Presented outcomes were the same as those used in previous evaluations and were chosen because there are few evidence-based and valid quality indicators for outpatient OA care [29]. Additional identified outcome indicators such as functional improvement and pain reduction [29] are not reflected in claims data. Among EUMUSC.net’s health-care quality indicators for OA [30], however, referral to specialists within three months can be observed in claims data. As we chose patients with ongoing OA, we decided against investigating referral times.

In terms of hospitalization, our results are consistent with findings based on Korean claims data showing that high continuity of care in patients with knee OA (operationalized using the Continuity of Care Index) is associated with lower relative risk of hospital admission [25].

In our study, patients in the intervention group were more likely to receive a prescription for physical therapy. A comparison with other programs is challenging because inclusion criteria used in other studies are not the same and health care provider referrals are handled differently [31]. In our study, for example, referrals could be provided by both GPs and orthopedists. Nonetheless, a prescription rate of 49.4% in a study cohort that subsequently received total joint replacement has been reported in Germany [28]. This is consistent with our findings in usual care, although ours did not necessarily precede total joint replacement.

In terms of obesity rates and other major potential risk factors [22, 32] with the exception of depression (34.3% in intervention group vs. 21.9% in control), the two study groups were similar at the outset. Furthermore, CCI was higher in the intervention group (2.2 vs. 1.6). This may reflect higher coding quality resulting from financial incentives, or simply higher prevalence in the intervention group, whereby the latter would underline the positive effects of the program.

Surgical procedure rates were lower in the intervention group, indicating that patients hospitalized as part of the orthopedic care program were less likely to undergo surgery. As the number of hospitalized patients was low, we decided against performing multivariable analysis and were therefore unable to adjust for influencing factors.

Prior studies have reported surgical procedure rates of 4–5% [33], which is similar to the rates we found in usual care. However, rates of endoprosthetic surgery were lower in collaborative orthopedic care (1.3%). It should be borne in mind that eligible patients were younger in the intervention group, and that both study groups were younger than the age cohort with the highest surgical procedure rates [33].

The likelihood of participation in exercise interventions was higher in the intervention group, which may have been the consequence of extended consultations [34]. As exercise is considered a major element of conservative OA therapy [35], a participation rate of 5.1% remains low and exercise interventions should receive greater support.

International experience of the successful implementation of structured and beneficial health care programs for OA patients indicates various challenges, such as high non-adherence rates in OA patients [36] or poorly integrated OA services within the community [16]. An example of an OA program is “Getting a Grip on Arthritis” [37], a multifaceted integrated client-centered training program for the management of arthritis in primary care in Canada. The aim of “Getting a Grip on Arthritis” is to implement clinical OA guidelines in primary care. The program therefore focuses mainly on providing the patient with information on OA and available community services. As a result, the number of patients provided with information almost doubled [37]. Improvement in medical care for OA patients has also been achieved through the use of follow-up support measures that are similar to those developed during regular clinical peer group meetings in the orthopedic care program. A further collaborative orthopedic care program in the USA was based on the chronic care model and has also proven beneficial to OA outpatients [38]. Like the orthopedic program in our study, this program also foresees longer consultations. Our orthopedic care program recommends consultation times of 20 min for basic disease assessments, compared with an unspecified consultation time in usual care. The extra time may be used to educate and inform patients, thus providing beneficial effects in terms of OA health literacy [34].

The major strengths of the study are the use of real-world data from multiple health care sectors and the large number of patients included in the analysis. This, in turn, allowed us to perform advanced statistical modelling. The review period was chosen after the program had been implemented for almost two years. It can therefore be assumed that the program had been fully implemented when our observations took place. Furthermore, the observation period of six quartiles minimizes seasonal effects in health care utilization, as well as bias resulting from changes in coding regulations. Additional benefits stem from the avoidance of contamination of control and intervention groups resulting from the exclusion of non-enrolled patients that consulted an enrolled orthopedist. Correspondingly, we also excluded patients that were enrolled in the program but had consulted an orthopedist that was not. The high participation of orthopedists in the program may explain the relatively few observations in the control group. Additionally, to be eligible for the control group, patients were not permitted to have enrolled in both the orthopedic care and GP-centered care programs, but to have consulted an orthopedist offering usual care. Nonetheless, it is impossible to determine whether the observed effects reflected enhanced orthopedic, or enhanced primary care.

The limitations of secondary data analysis based on insurance claims data have been described in previous evaluation studies [9, 12]. The accuracy of our analysis is dependent on the quality of claims data, such as coding quality, data availability and data transfer. Clinical information, pain levels, functional impairment and information on patient preferences were not available. Furthermore, despite an extensive set of covariables, bias due to unmeasured confounders cannot be ruled out. For physical therapy, it should be noted that we did not differentiate between physical therapy prior to or post replacement surgery, as we did not have data before 2014, and surgical procedure rates in the observation period were low.

Since patients and doctors participate in the program voluntarily, we cannot rule out self-selection bias in both GP-centered and orthopedic care. Patients that enrolled in the program may initially be more adherent and consequently more likely to benefit from consultation-oriented therapeutic strategies. It is further possible that the intrinsic motivation behind the decision of GPs and orthopedists to participate in the health care programs resulted in improved health-care. It is possible that participation in the collaborative program itself produced a bias towards prolonged conservative treatment in spite of no improvement in symptoms. It is even possible that orthopedists that favor conservative treatment actively discourage patients from arthroplasty. Physician visits could not be assessed since physicians are reimbursed per quartile, irrespective of the number of visits. Furthermore, medication could not be assessed specifically for OA. Additionally, as we did not analyze clinical data and do not know what occurred during consultations, it is difficult to assess what effects contributed directly to our observations. However, we can obtain an idea of the enhancement of conservative outpatient therapies by observing increased opportunities for physical therapy and exercise interventions. Hence, qualitative studies aimed at assessing and, if possible, measuring the effects of consultations, along with randomized controlled trials, may provide further insights.

## Conclusion

Collaborative ambulatory orthopedic care is a unique health-care program that promotes collaboration between primary and specialist health care providers in the care of patients with musculoskeletal disorders. In our observational cohort study, we discovered that in program participants, the likelihood that enhanced conservative therapies had been pursued (e.g. physical therapy and exercise interventions) increased, while the risk of OA-related hospitalization decreased. The sustainability of the program should be further investigated.

## Data Availability

The data that support the findings of this study are available from AOK Baden-Wurttemberg but restrictions apply to the availability of these data, which were used under license for the current study, and so are not publicly available. Data are however available from the authors upon reasonable request and with permission of AOK Baden-Wurttemberg.

## Abbreviations

AOK: ‘Allgemeine Ortskrankenkasse’
CCI: Charlson Comorbidity Index
CI: Confidence interval
OA: Osteoarthritis
OR: Odds Ratio
RR: Rate Ratio
